# Detail Preserving Low Illumination Image and Video Enhancement Algorithm Based on Dark Channel Prior

**DOI:** 10.3390/s22010085

**Published:** 2021-12-23

**Authors:** Lingli Guo, Zhenhong Jia, Jie Yang, Nikola K. Kasabov

**Affiliations:** 1 College of Information Science and Engineering, Xinjiang University, Urumqi 830046, China; gll@stu.xju.edu.cn; 2 Institute of Image Processing and Pattern Recognition, Shanghai Jiao Tong University, Shanghai 200400, China; jieyang@sjtu.edu.cn; 3 Knowledge Engineering and Discovery Research Institute, Auckland University of Technology, Auckland 1020, New Zealand; nkasabov@aut.ac.nz; 4 Intelligent Systems Research Center, Ulster University Magee Campus, Derry BT48 7JL, UK

**Keywords:** dark channel prior, image detail preserving, low illumination, images, video

## Abstract

In low illumination situations, insufficient light in the monitoring device results in poor visibility of effective information, which cannot meet practical applications. To overcome the above problems, a detail preserving low illumination video image enhancement algorithm based on dark channel prior is proposed in this paper. First, a dark channel refinement method is proposed, which is defined by imposing a structure prior to the initial dark channel to improve the image brightness. Second, an anisotropic guided filter (AnisGF) is used to refine the transmission, which preserves the edges of the image. Finally, a detail enhancement algorithm is proposed to avoid the problem of insufficient detail in the initial enhancement image. To avoid video flicker, the next video frames are enhanced based on the brightness of the first enhanced frame. Qualitative and quantitative analysis shows that the proposed algorithm is superior to the contrast algorithm, in which the proposed algorithm ranks first in average gradient, edge intensity, contrast, and patch-based contrast quality index. It can be effectively applied to the enhancement of surveillance video images and for wider computer vision applications.

## 1. Introduction

With the development of science and technology, surveillance video plays an important role in the field of public safety. However, in low light conditions at night, the light entering the video device is insufficient, which results in a bad visual effect on the recorded video. These negative effects may include low brightness and contrast, color distortion, and poor visibility. To make better use of videos captured in low illumination, we have to enhance them.

Videos are composed of multiple single-frame images presented in time, so video enhancement can be achieved by enhancing each low illumination image. Many enhancement algorithms have been proposed based on certain characteristics of low illumination images. To improve the brightness of low illumination images, some methods directly modified the size and distribution of pixels, such as gray transformation methods and histogram equalization (HE) methods [1,2]. The grayscale transformation algorithm improved the brightness of the image by increasing the pixel value. However, the algorithm has poor adaptability and limited enhancement ability, so it is often used in combination with other algorithms, the most common of which are gamma transformation [3] and log transformation [4]. And the algorithm based on histogram equalization [5] is prone to color distortion. Therefore, researchers proposed a series of enhancement algorithms based on Retinex [6] to avoid the problem of directly adjusting the pixel values. For example, the multiscale color recovery Retinex algorithm (MSRCR) [7] considered color fidelity, however, this method only served reflection beforehand, making the enhancement result unnatural. So, the subsequent Retinex combined illumination with reflectance as an enhancement achieved a good effect [8,9]. However, the algorithm is prone to the phenomenon of insufficient/excessive enhancement, which leads to the loss of image details.

In recent years, researchers have experimented with other ways to enhance low illumination images. By using similarity between low illumination inverted images and hazy images, many researchers have applied the dehazing algorithm [10] to enhance low illumination images. However, similar to all algorithms mentioned above, this algorithm also has the following problems:

(1) The enhancement results obtained by the algorithm contain less detailed information and cannot effectively highlight the key information in the image.

(2) The edge retention ability of the enhanced image obtained by the algorithm is poor, especially in brighter areas, and the image edge is blurred due to the phenomenon of excessive enhancement.

(3) The brightness of the enhanced image obtained by some dehazing algorithms is insufficient, and the light source is prone to overexposure, which results in the loss of image information.

To solve the problems of the existing algorithms, a detail preserving low illumination video image enhancement algorithm based on dark channel prior is proposed. First, the low illumination image is inverted to obtain the dark channel, and then refine the dark channel to improve the brightness of the low illumination image. Second, the global atmospheric light and transmission are obtained from the refined dark channel, and then the transmission is refined by using an anisotropic guided filter. Third, the refined transmission and atmospheric light are substituted into the atmospheric scattering model to obtain a clear image after dehazing, and then the clear image is reversed to obtain the initial enhanced image. Finally, due to the insufficient details of the initial enhanced image, a detail enhancement method is proposed, and the high frequency of the initial enhanced image is added to itself. According to the low illumination image characteristics, the darker the area in the image, the worse the visibility of details. Therefore, an S-type function is defined as the high-frequency factor of the addition by using the visibility function, and the final enhancement result is obtained.

Compared with existing algorithms, the main contributions of the proposed algorithm in this paper are as follows:

(1) The dark channel is refined by imposing a structure prior to the initial dark channel to obtain a well-structured dark channel map, which can better enhance the brightness of low illumination images to achieve a better enhancement effect.

(2) Because local processing produces a blocking effect, it is necessary to refine the initial roughly estimated transmission. Considering the edge-preservation problem, the anisotropic guided filter is used instead of the original soft-matting refined algorithm to better preserve the edges and significantly reduce the processing time.

(3) According to a large number of experiments, the details of the enhancement results obtained by dehazing algorithms are insufficient. Therefore, a detail enhancement method is proposed, which adds the high frequency of the initial enhanced image to itself. An S-type function is defined as the high-frequency factor of the addition, which is based on the low illumination image characteristics; that is, the darker areas in the image have poorer detail visibility. The details and clarity of the low illumination video image are improved better by the step.

(4) For video enhancement, a large difference in the brightness of each frame enhanced image will cause the composite video to flicker. To avoid this, subsequent video frames are adjusted based on the average brightness of the first enhanced frame to control the enhancement of each frame and reduce the brightness difference between the front and back frames.

The results of qualitative and quantitative evaluations show that our method can significantly improve the video captured in low illumination conditions and has better results than other methods. It preserves the details in the original images.

The remainder of this paper is organized as follows. After describing the related work in Section 2, the proposed algorithm is presented in Section 3. Along with presenting comparative results, Section 4 evaluates the performance of the proposed algorithm. Discussion on the method is presented in Section 5 and conclusion in Section 6.

## 2. Related Work

At present, many algorithms for low illumination image and video enhancement have been proposed and can be roughly divided into two categories: machine learning and traditional algorithms.

In recent years, with the rapid development of machine learning, an increasing number of researchers have applied it to video image processing [11,12,13,14,15]. Based on the theory of traditional algorithms, some learning models have been created. For example, Zhao et al. [11] proposed a multi-path interactive network to enhance color images for more natural enhancement results. Kim et al. [16] built a Low-LightGAN using a generative antagonistic network, whose training images were produced by local illumination. Zhang et al. [17] enhanced low illumination images in CIELAB space, combining deep convolution and generation of antagonistic networks to more accurately estimate the illumination map. The main advantages of machine learning over traditional enhancement algorithms are that they are easier to train on new data and achieve better performance. However, such methods require the support of large datasets, and images with good contrast corresponding to low illumination video images are not easily available. In addition, the time complexity of the algorithms increases with the complexity of the model. Therefore, many scholars have performed innovative work on traditional low illumination video image enhancement algorithms. For video enhancement, there are three types of algorithms [18,19]. The first one is to divide the video into frames and synthesize the video after each frame image is processed. The second method divided the video into foreground and background, and only enhanced the background. The third approach is similar to the first, except that it takes into account the correlation of adjacent frames, thus avoiding adverse phenomena such as flickering in the synthesized video.

The first type of algorithms essentially enhanced a single image, including the Retinex-based algorithm, the fusion-based algorithm, and the dehazing model-based algorithm, etc. Many algorithms have been proposed based on the Retinex theory. Early Retinex algorithms, such as single-scale Retinex (SSR) [20], multiscale Retinex (MSR) [21], and the multiscale Retinex algorithm with color restoration (MSRCR) [7,22], directly used the reflectance map as an enhancement map, resulting in unnatural results and over enhancement. Therefore, in subsequent algorithms, the illumination map was compensated to the reflectivity to obtain an enhanced image. For example, Guo et al. [23] proposed low light image enhancement via illumination map estimation (LIME), which perfected the initial illumination by imposing a structure on the initial illumination. However, the algorithm is prone to overexposure and noise amplification. To solve the noise problem, Hao et al. [24] added a regularization term to the reflectance to suppress the imaging noise. Retinex algorithms have advantages for color image enhancement, but halos easily appear at the edge of the enhanced image, and some results are too bright, losing details.

Recent fusion-based algorithms are often based on single-image fusion. Ren et al. [25] combined the camera response model (CRM) and Retinex to obtain enhanced images with less brightness and color distortion by adjusting low illumination images’ exposure. Li et al. [26] proposed low illumination video image enhancement based on wavelet fusion, which uses wavelet fusion in RGB space after increasing the brightness of the same frame image 10 times. Fusion-based methods can effectively enhance the brightness of the image, but this model is prone to over-enhancement and loss of detail.

Using a relationship between low illumination images and hazy images, researchers proposed a low illumination image enhancement algorithm based on the dehazing theory. Kaiming He first proposed the dark channel prior concept [27], which is widely used in the image field. Pang et al. [28] improved the dark channel dehazing algorithm by introducing gamma correction to improve image contrast. Recently, Wang et al. [29] proposed the absorption light scattering model (ALSM), which reasonably explained the imaging process of low illumination images and obtained good enhancement results. However, the algorithm based on the dehazing model has some disadvantages, such as insufficient enhancement of dark areas, easy overexposure of bright areas, poor detail, and edge preservation.

The second type of algorithms can be divided into two categories according to whether or not a video with good daylight illumination is required. Rao et al. [30] proposed fusing night video and daytime background brightness in a gradient-domain to obtain enhanced images. Soumya et al. [31] used wavelet fusion to fuse night video frames with daytime background frames to effectively highlight the illumination area. Lee et al. [32] divided the video frame into the dark target area and bright background area and then fused the adjusted dark area with the bright area to enhance the video. Because these algorithms only adjust the background area of an image, they can effectively preserve image details and edge information. However, if the background image is not extracted accurately, the enhanced image may be distorted.

The third method incorporates specific processing to make the enhanced video more natural. To save video processing time, Dong et al. [33] used the same transmission for similar frames, which also avoided to some extent video flickering. Zhang et al. [34] used spatiotemporal filtering to eliminate video noise and avoid flicker artifacts. Ko et al. [35] proposed an algorithm to recover low light video using similar blocks between time adjacent frames, using average brightness and improved color allocation to reduce enhanced video color distortion and flicker. Zhang et al. [36] proposed a video enhancement method based on region system and image fusion, which maintains temporal consistency by propagating zone regions from the previous frame to the current frame. Buades et al. [37] proposed a denoising algorithm for real video scenes. Using the self-similarity and redundancy of adjacent frames, motion compensation is used to stabilize video sequences by regularized optical flow method. Ren et al. [38] proposed the LR3M model, which was the first to add a low-rank decomposition model in the decomposition process. It can remove noise from low illumination images very well, but the algorithm is more complex and may blur the image details. When processing video, the illumination consistency between frames was enhanced to reduce video flicker by introducing a coherence term.

Aiming at overcoming the shortcomings of the existing algorithms, our proposed method offers new features, such as

(1) Low illumination video images in different scenes are considered, including overall darker video images, local brighter video images, and video images with light sources. The proposed algorithm is tested in these scenes. The results show that the proposed algorithm can better balance the degree of brightness enhancement and edge detail retention, suppress overexposure to a certain extent, and obtain more natural enhancement results. The proposed algorithm can be applied to different low light scenes and has wider applicability.

(2) The dehazing algorithm is improved based on the dark channel to be applied to low illumination video image enhancement. A dark channel refining method is proposed to improve the brightness of low illumination images. The anisotropic guided filter is used to refine the transmission, resulting in a finer transmission and better preservation of the edges and details of the enhanced image.

(3) Detail enhancement method is proposed. By defining an S-type function as the coefficients of the high-frequency part, the high-frequency part is added to the initial enhanced image to obtain the enhanced image with prominent detail and better visual effect.

(4) Subsequent video frames brightness is adjusted based on the average brightness of the first frame to make the average brightness of the video frame sequence consistent, thereby reducing flickering.

## 3. Proposed Method

**Figure 1:**
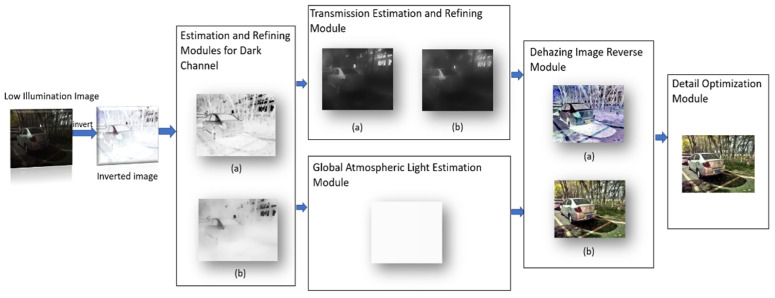
Flowchart of the proposed method. Among it, in the dark channel estimation and refinement module (**a**) is the dark channel estimation result, (**b**) is the dark channel refinement result; in the transmission estimation and refinement module (**a**) is the transmission estimation result, (**b**) is the transmission refinement result; in the dehazing image reverse module (**a**) is the dehazing image, and (**b**) is the initial enhanced image obtained by reverse.

In this section, based on the similarity between low illumination inverted images and hazy images, a detail preserving low illumination video image enhancement algorithm based on dark channel prior is proposed to enhance the video images captured in low illumination conditions. First, invert the low illumination image, calculate the dark channel of the inverted image, and refine the dark channel by imposing a structure prior to obtaining a well-structured dark channel map, which better enhances the brightness of the low illumination image. Second, the atmospheric light and transmission are obtained from the refined dark channel, and the transmission is refined by the anisotropic guide filter. Then, based on the inversion of the results from the atmospheric scattering model, an initial enhanced image is obtained. Finally, the initial enhanced image is optimized for details, and the final enhanced image with good details is obtained. The flowchart of the proposed method is shown in Figure 1.

### 3.1. Atmospheric Scattering Model

The classic atmospheric scattering model [39] is described as

(1) I(x)=J(x)t(x)+A(1−t(x))

where I(x) is a hazy image, J(x) is a scene radiance, A is the global atmospheric light, and t(x) is the medium transmission describing the portion of the light that is not scattered and reaches the camera.

In this paper, hazy image I(x) is the inversion of low illumination image M(x), defined as follows:

(2) I(x)=1−M(x)

### 3.2. Dark Channel and Its Prosed Modification

#### 3.2.1. Basic Concept of Dark Channel

For a color image F(x), at least one-color channel has some pixel that tends to be zero, which is called the dark channel. It can be defined as follows:

(3) Fdark(x)= yϵΩ(x)min( cϵ{r,g,b}minFc(y))

If F is a haze-free image, except for the sky region, the intensity of F’s dark channel is low and tends to be zero $F^{dark}\left(x\right)\to 0$. Conversely, if F is a hazy image, its dark channel does not tend to zero. Shadow portions, dark objects, and low reflectivity objects in the image are all important factors contributing to the existence of dark channels.

**Figure 2:**
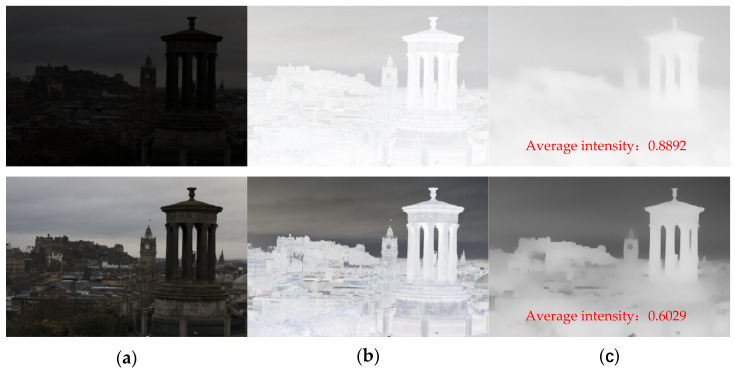
Relationship between low illumination image and its dark channel image. (**a**) Images at different exposure levels. (**b**) Inverse image corresponding to (**a**). (**c**) Dark channel map corresponding to (**b**).

By observing the hazy image, it can be seen that the brightness of the hazy image is higher than that of the haze-free image because of the additional light effect. Therefore, the intensity of the dark channel is higher where the haze is denser. For inverted low illumination images, the lower the illumination, the higher the haze of the inverted image, and the higher the intensity of the dark channel, as shown in Figure 2.

#### 3.2.2. Proposed Refined Method for Dark Channel

Dark channel maps of low-illumination inverted images are the basic layer of low illumination inverted images, so dark channel maps should be as smooth as possible and keep the overall structure of the image. However, the initial dark channel map obtained by minimizing operation does not conform to this concept, so the dark channel is refined by imposing a structure on the initial dark channel to obtain a well-structured dark channel map. Based on the initial dark channel $I^{dark}$, we solve the following optimization problem:

(4)  I^darkmin∥Idark−I^dark∥F2+α∥W·∇I^dark∥1

where α is a custom factor used to balance the correlated terms in Formula (4), and ${∥\cdot ∥}_{F}$ and ${∥\cdot ∥}_{1}$ designate the Frobenius and $\ell_{1}$ norms. In addition, W is the weight matrix, and $\nabla {\hat{I}}^{dark}$ is the first derivative of ${\hat{I}}^{dark}$ in the horizontal ($\nabla_{h}{\hat{I}}^{dark}$) and vertical ($\nabla_{v}{\hat{I}}^{dark}$) directions. In the objective (4), the first term considers the fidelity between the initial dark channel $I^{dark}$ and its refined channel ${\hat{I}}^{dark}$, and the second considers the smoothness.

In problem (4), a fast algorithm can be used to solve the problem without iteration, which greatly improves the processing speed. The second term $∥W\cdot \nabla {\hat{I}}^{dark}∥{}_{1}$, which contains the $\ell_{1}$ norm and derivation, is the more complex term. To simplify the operation, the following formula can be used:

(5) $$\underset{\varepsilon \to 0^{+}}{lim}\sum_{x}\sum_{d\epsilon \left\{h,v\right\}}\frac{W_{d}\left(x\right){\left(\nabla_{d}{\hat{I}}^{dark}\left(x\right)\right)}^{2}}{\left|\nabla_{d}I^{dark}\left(x\right)\right|+\varepsilon }=∥W\cdot \nabla {\hat{I}}^{dark}∥{}_{1}$$

Based on the above formula, $\sum_{x}\sum_{d\epsilon \left\{h,v\right\}}\frac{W_{d}\left(x\right){\left(\nabla_{d}{\hat{I}}^{dark}\left(x\right)\right)}^{2}}{\left|\nabla_{d}I^{dark}\left(x\right)\right|+\varepsilon }$ can be used instead of $∥W\cdot \nabla {\hat{I}}^{dark}∥{}_{1}$ to rewrite problem (4) as follows:

(6)  I^darkmin∥Idark−I^dark∥F2+α∑x∑dϵ{h,v}Wd(x)(∇dI^dark(x))2|∇dIdark(x)|+ε

Similar to the original function, the objective function after transformation is to extract the structure of the initial dark channel $I^{dark}$. Specifically, when the gradient of the initial dark channel $\left|\nabla_{d}I^{dark}\left(x\right)\right|$ is small, the target gradient is also suppressed. In contrast, when the gradient of the initial dark channel $\left|\nabla_{d}I^{dark}\left(x\right)\right|$ is strong, the suppression is alleviated. In this way, the edge of the dark channel map can be effectively kept.

Problem (6) only involves quadratic terms. Therefore, the problem can be solved directly by solving the following:

(7) $$\left(E+\sum_{d\in \left\{u,v\right\}}D_{d}^{T}Diag\left({\tilde{w}}_{d}\right)D_{d}\right){\hat{i}}^{dark}=i^{dark}$$

where E is the identity matrix, ${\tilde{w}}_{d}$ is the vector representation of ${\tilde{W}}_{d}$, and ${\tilde{W}}_{d}\leftarrow \frac{W_{d}\left(x\right)}{\left|\nabla_{d}I^{dark}\left(x\right)\right|+\varepsilon }$. $Diag\left({\tilde{w}}_{d}\right)$ is a diagonal matrix composed of ${\tilde{w}}_{d}$.

The weight of each position is defined as follows:

(8) $$W_{h}\left(x\right)\leftarrow \sum_{y\epsilon \Omega \left(x\right)}\frac{G_{\sigma }\left(x,y\right)}{\left|\sum_{y\epsilon \Omega \left(x\right)}G_{\sigma }\left(x,y\right)\nabla_{h}I^{dark}\left(y\right)\right|+\varepsilon }\;W_{v}\left(x\right)\leftarrow \sum_{y\epsilon \Omega \left(x\right)}\frac{G_{\sigma }\left(x,y\right)}{\left|\sum_{y\epsilon \Omega \left(x\right)}G_{\sigma }\left(x,y\right)\nabla_{v}I^{dark}\left(y\right)\right|+\varepsilon }$$

where $G_{\sigma }\left(x,y\right)$ is generated by a Gaussian kernel function with standard deviation σ. $G_{\sigma }\left(x,y\right)$ is expressed in the following:

(9) $$G_{\sigma }\left(x,y\right)\propto exp\left(-\frac{dist\left(x,y\right)}{2\sigma^{2}}\right)$$

where dist(x,y) denotes the spatial Euclidean distance between x and y. Different from RTV, the weight matrix in Equation (8) is constructed based on the given $I^{dark}$ rather than updated iteratively according to ${\hat{I}}^{dark}$. Therefore, the weight here only needs to be calculated once.

### 3.3. Transmission and Its Refined Method

#### 3.3.1. Transmission Estimation

For any low illumination inverted image, select the top-1 percent brightest pixels in the refined dark channel. Among these pixels, the pixels with the highest intensity in the low illumination inverted image are selected as atmospheric light. Atmospheric light A is a three-element vector, and each element corresponds to each color channel. Normalized the atmospheric scattering model by A:

(10) $$\frac{I^{c}\left(x\right)}{A^{c}}=t\left(x\right)\frac{J^{c}\left(x\right)}{A^{c}}+1-t\left(x\right)$$

where normalization is performed individually for each color channel.

Assume that the transmission of each image block is constant. $\tilde{t}\left(x\right)$ is used to represent the initial transmission and then calculate the dark channels on both sides of the normalization equation. The minimal operation is put on both sides of the equation, and the following can be obtained:

(11)  yϵΩ(x)min( cϵ{r,g,b}minIc(y)Ac)=t˜(x) yϵΩ(x)min( cϵ{r,g,b}minJc(y)Ac)

Because $\tilde{t}\left(x\right)$ is a constant, it can be placed outside of the minimum operation.

The dark channel of the haze-free image is close to zero. Therefore, the dark channel of scene radiation J is close to zero, and the following is true:

(12) Jdark(x)= yϵΩ(x)min( cϵ{r,g,b}minJc(y))=0

Since the atmospheric light $A^{c}$ is positive, the following formula holds true:

(13)  yϵΩ(x)min( cϵ{r,g,b}minJc(y)Ac)=0

Substituting (13) into (10) eliminates the multiplication term and obtains the following results:

(14) t˜(x)=1− yϵΩ(x)min( cϵ{r,g,b}minIc(y)Ac)

where  yϵΩ(x)min( cminIc(y)Ac) is a refined dark channel of the normalized low illumination inverted image. It can be seen in (14) that the transmission is only related to the refined dark channel.

A weighting factor ω is introduced to control the degree of enhancement, resulting in the initial transmission as follows:

(15) t˜(x)=1−ω yϵΩ(x)min( cϵ{r,g,b}minIc(y)Ac)

The default ω value for this paper is 0.95.

#### 3.3.2. Proposed Refined Method for Transmission

Because the estimated $\tilde{t}\left(x\right)$ is not continuous at the local block boundary, further refinement of $\tilde{t}\left(x\right)$ is required. In this paper, anisotropic guided filters are used to smooth the transmission map while preserving its edges.

Aiming at the problems of detail halos in the results obtained by guide filters [40] and poor performance in handling inconsistent structures between guided and input image blocks, Carlo et al. proposed an anisotropic filter (AnisGF) [41], which integrates anisotropy into the filter formula to better preserve the edge details of the image. A brief introduction to anisotropic filters is given below with more information in the reference.

Unlike guided filters, anisotropic guided filters use weighted averaging to achieve maximum diffusion. By introducing the weight factor $\omega_{i,j}$, the scaling factor $a_{j}$, and the deviation factor $b_{j}$ are weighted and the following results are obtained:

(16) $$\tilde{a_{i}}=\sum_{j\epsilon N\left(i\right)}\omega_{i,j}a_{j}\;\tilde{b_{i}}=\sum_{j\epsilon N\left(i\right)}\omega_{i,j}b_{j}$$

where $\omega_{i,j}$ is defined as the weight assigned to pixel j around center pixel i.

To achieve maximum diffusion while preserving strong edge boundaries in the guide image, the weight is designed. Because the maximum diffusion is achieved when $a_{i}\to 0$, the objective function can be obtained as follows:

(17)  wiargmina˜i2+μ∑jϵN(i)∥ωi,j∇j∥22

where $w_{i}$ is a weight vector for all neighborhoods centered on pixel i, and $\nabla_{j}$ is the gradient vector contained in the neighborhood of j in the guide image.

The above formula is simplified to obtain the following weight solution:

(18) $$\omega_{i,j}=\omega_{j}=\frac{\epsilon }{\sigma_{gj}^{2\alpha }+\epsilon }$$

The final weighted parameters and output are obtained by normalizing the weighted solution:

(19) $${\tilde{a}}_{i}=\frac{\sum_{j\epsilon N\left(i\right)}\omega_{i,j}a_{j}}{\sum_{j\epsilon N\left(i\right)}\omega_{i,j}}\;{\tilde{b}}_{i}=\frac{\sum_{j\epsilon N\left(i\right)}\omega_{i,j}b_{j}}{\sum_{j\epsilon N\left(i\right)}\omega_{i,j}}\;{\hat{x}}_{i}={\tilde{a}}_{i}g_{i}+{\tilde{b}}_{i}$$

where $g_{i}$ is the guidance image and ${\hat{x}}_{i}$ is the filtered output image. In this paper, the input image is the transmission, the guide image is the gray image of the low illumination inversion image, and the output is the refined transmission. Compared with the soft-matting algorithm, the anisotropic guided filter has a better edge-preserving effect and faster speed. Compared with the fast enhancement algorithm using a guided filter, it has a better edge-preserving effect. The specific results are shown in the experimental section.

The atmospheric light value and the refined transmission are substituted into the atmospheric scattering model to obtain a clear image after dehazing. However, when the refined transmission t(x) tends to zero, J(x)t(x)→0, and the directly recovered J(x) easily produces noise. Therefore, set the lower bound of the transmission t(x) to $t_{0}$, and the expression of the dehazing image is as follows:

(20) $$J\left(x\right)=\frac{I\left(x\right)-A}{max\left(t\left(x\right),t_{0}\right)}+A$$

In this paper, the value of $t_{0}$ is 0.1. The initial enhanced image N(x) is the inversion of clear image J(x):

(21) N(x)=1−J(x)

### 3.4. Proposed Alternative Scheme for Video Detail Enhancement

After a large number of experiments, it is found that the details of the enhanced image obtained by the improved algorithm based on dark channel dehazing are insufficient, so the detail enhancement module is introduced to improve the details of the enhanced image.

According to the characteristics of low illumination images, the darker areas in the image contain less detailed information, and the detail is generally reflected in the high-frequency part of the image. Therefore, in the case of insufficient detail information, the high-frequency part of the image can be extracted and added to the image. Considering the rich detail and high visibility of bright areas in images, direct addition of high frequency may result in too many details, so an S-type function is defined as the coefficient of addition of high frequencies using the visibility function. The coefficient function is defined as follows:

(22) $$A\left(x,y\right)=\frac{1}{1+e^{-\left(vis\left(x,y\right)-vis_{m}\right)/k}}$$

where k is used to control the slope of the function; in this paper, the value is 0.01. vis is a visibility function, and $vis_{m}$ is mean value. It can be defined as follows:

(23) $$vis\left(x,y\right)=\frac{\Delta L\left(x,y\right)}{L_{B}\left(x,y\right)}=\frac{L\left(x,y\right)-L_{B}\left(x,y\right)}{L_{B}\left(x,y\right)}$$

In the above formula, L(x, y) is the value component in HSV space, and $L_{B}\left(x,y\right)$ is the result of a Gaussian filter [42] on the value component, called the background brightness component.

The final enhanced image can be expressed as

(24) R(x,y)=N(x,y)+A(x,y)Hig(x,y)

where Hig(x,y) is the high-frequency of the initial enhanced image.

## 4. Experimental Results and Analysis

In this section, the multi-exposure image pairs and video datasets [43], and low-illumination video images were taken in the field are used to test. The size of the multi-exposure image is 1200 × 800, the size of the video frame is 720 × 480 and 1280 × 720, and the time lengths are different. The size of the test image we took was 576 × 432, and the length of the test video was different, the size of each frame was 1920 × 1080. Through the qualitative and quantitative analysis of the test results, the proposed method is compared with other methods comprehensively. Other methods include the LIME algorithm, the ALSM algorithm, Li et al. [26] based on the wavelet fusion algorithm, and the LR3M algorithm. The comparative experiment in this paper consists of two parts. The first part compares the result of the dark channel prior dehazing algorithm with that of the proposed algorithm. The second part compares the algorithm proposed in this paper with other algorithms through qualitative and quantitative analysis. To demonstrate the effectiveness of the proposed methods, all compared methods are implemented in MATLAB 2019a on an Intel Core i5 3.20-GHz processor with 4 GB RAM, running a Windows 10 operating system.

### 4.1. Parameter Setting

**Figure 3:**
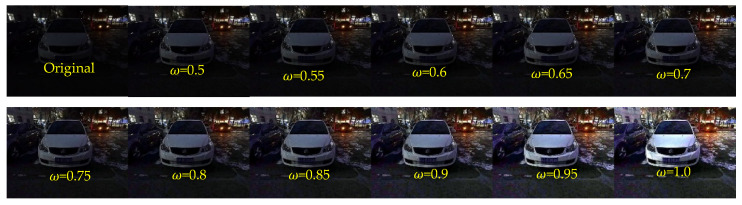
Influence of different values of the parameter ω on the enhancement results.

In this paper, the parameters ω and $t_{0}$ influence the enhancement effect. The parameter ω controls the degree of enhancement of the image, ωϵ[0, 1]. With the increase of ω, the brightness of the image can be improved better. However, when ω=1, there will be severe overexposure in some areas of the image. Therefore, make ω=0.95 obtain appropriate enhancement results. The specific assignment process is shown in Figure 3.

**Figure 4:**
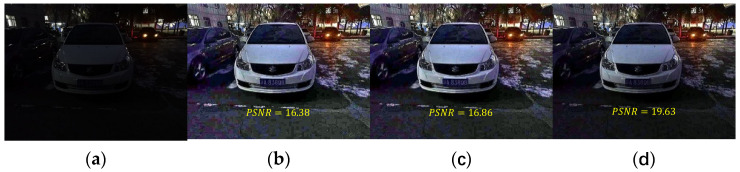
Selection of parameter $t_{0}$.(**a**) original image. The enhancement result with (**b**) $t_{0}$ not to be introduced, (**c**) $t_{0}=0.1$, and (**d**)$\;t_{0}=0.2$.

In this paper, a small parameter $t_{0}$ is introduced to reduce the noise of the enhanced image. Compared the enhancement results without introducing $t_{0}$ and taking different values of $t_{0}$, and find that the ground noise amplification is obvious without introducing $t_{0}$, and the brightness of the enhanced image is insufficient when $t_{0}=0.2$, so take the classical value of 0.1 to get the result of moderate brightness and noise. The specific results are shown in Figure 4.

### 4.2. Ablation Experiment

The proposed algorithm is continuously improved based on the dark channel prior dehazing algorithm, so several aspects of improvement are experimented and analyzed.

#### 4.2.1. Comparative Analysis of Transmission Refined Method

**Figure 5:**
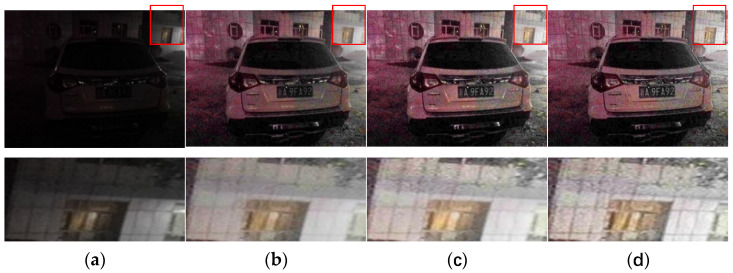
(**a**) Test image and its detail map; (**b**) soft-matting refined algorithm result and its detail map; (**c**) guided filter refined algorithm result and its detail map; (**d**) AnisGF refined algorithm result and its detail map.

The results obtained by our refined method (AnisGF) are compared with those obtained by the original soft-matting refined algorithm and the guided filter refined algorithm. The results are shown in Figure 5.

From the results in the figure, it can be seen that compared with the soft-matting algorithm, the enhanced results obtained by the guided filter and the AnisGF are brighter, and the key information such as the license plate is clearer. This may be because the guided filter used the grayscale image of the low illumination inverted image as the guided image, which makes the output brighter. The edge details of the enhanced image obtained by the AnisGF used in this paper are better preserved than those obtained by the guided filter, which can be seen from the detailed map of the enhanced image. This is because AnisGF introduced anisotropic diffusion into the filter. Anisotropic diffusion determines whether to diffuse the surrounding pixels based on the relationship between the current pixel and the surrounding pixels. When a neighborhood pixel differs greatly from the current pixel, the neighborhood is probably the boundary, and the current pixel does not diffuse in that direction, thus preserving the boundary.

#### 4.2.2. Comparative Analysis before and after Dark Channel Refinement

**Figure 6:**
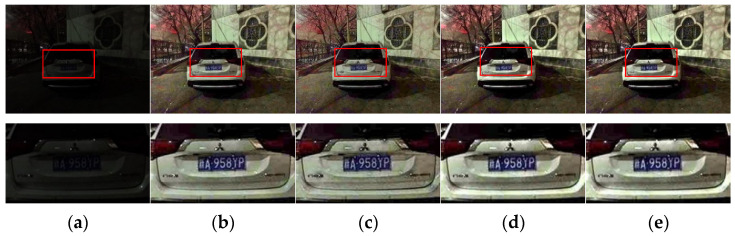
(**a**) Test image and its detail map, (**b**) guided filter refining transmission result and its detail map, (**c**) AnisGF refining transmission result and its detail map, (**d**) guided filter refining transmission + dark channel refining result and its detail map, and (**e**) AnisGF refining transmission + dark channel refining result and its detail map.

Since the above proves that the enhancement results obtained by the guided filter and AnisGF are good for transmission refinement, this section only applied dark channel refinement to the comparison of the two algorithms to illustrate the necessity of this step, and the result is shown in Figure 6.

As seen in the results in the diagram, the enhanced result with dark channel refinement is brighter and clearer. Specifically, for the results of AniGF refining transmission, dark channel refining makes the enhanced image more natural. It can be known from the above that AnisGF preserves edges well, but for some images (such as the test image in this section), it causes black shadows around the light spots on the car. However, when dark channel refining is added, the shadows disappear, making the image more in line with the visual characteristics of the human. For this reason, dark channel refinement may improve the structure of the obtained dark channel, and the initial transmission is obtained from the dark channel, so the final transmission obtained by the dark channel refinement step is more accurate; thus, a better enhancement effect is obtained.

#### 4.2.3. Comparative Analysis before and after Detail Enhancement

**Figure 7:**
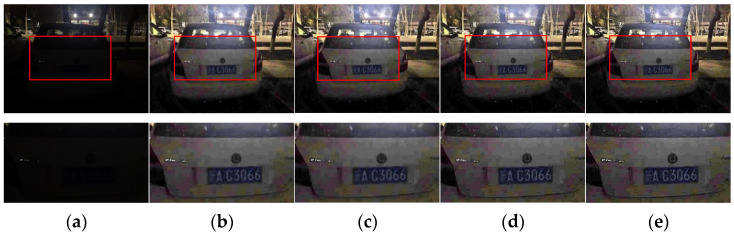
(**a**) Test image and its detail map, (**b**) guided filter refining transmission + dark channel refining result and its detail map, (**c**) AnisGF refining transmission + dark channel refining result and its detail map, (**d**) guided filter refining transmission + dark channel refining + detail enhancement result and its detail map, and (**e**) AnisGF refining transmission + dark channel refining + detail enhancement result and its detail map.

For some images, the result enhanced by the improved dehazing method has insufficient detail and low clarity, so the detail enhancement was added. To prove the necessity of this step, the following experiments and analyses are performed, and the results are shown in Figure 7.

As seen in the results, the details of the image obtained by using detail enhancement are more prominent, and the car logo and the numbers in the license plate are more prominent in the image, which is more in line with the license plate seen by the human eye. It can also be seen that the overexposure of the algorithm at the light source is less, and the difference between the size of the light source in the enhanced image and that in the original image is smaller. From this, it can be concluded that the detail enhancement part can effectively enhance the details in the dark of the image, and this step is also necessary.

After the above three groups of experiments, the effectiveness of the proposed detail preserving low illumination video image enhancement algorithm based on dark channel prior can be seen. The proposed algorithm performs well in darker images as a whole, in images with bright areas, and images with light sources. It effectively enhanced the brightness of low illumination images while maintaining the details and suppressing overexposure. Next, the superiority of the proposed algorithm is illustrated by comparing it with other algorithms.

### 4.3. Comparative Experiments and Analysis

#### 4.3.1. Quantitative Analysis

**Figure 8:**
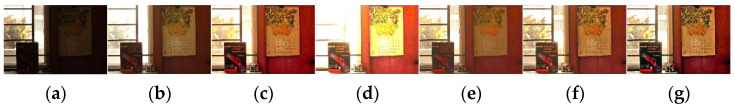
(**a**) Original image; (**b**) Reference. The remaining images are enhancement results, generated by the following methods: (**c**) ALSM, (**d**) Li et al. [26], (**e**) LR3M, (**f**) LIME, and (**g**) the proposed method.

**Figure 9:**
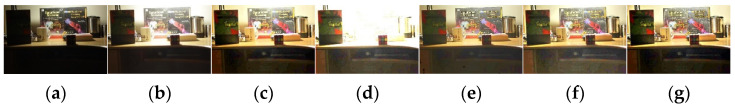
(**a**) Original image; (**b**) Reference. The remaining images are enhancement results, generated by the following methods: (**c**) ALSM, (**d**) Li et al. [26], (**e**) LR3M, (f) LIME, and (**g**) the proposed method.

Figure 8 and Figure 9 show the experimental results in multi-exposure data. It can be seen from the results that all enhancement algorithms are effective for low illumination images, but there are some problems with the enhancement results obtained by the comparison algorithms. Li et al. algorithm has severe overexposure, which overwhelms many details in the enhanced image. The ALSM algorithm does not preserve edge details well in bright background areas, as shown in Figure 8. The LR3M algorithm has an insufficient effect on brightness enhancement in dark areas and blurs key information such as text. The LIME algorithm and the proposed algorithm show better performance, but the enhanced image by the proposed has clearer detail information.

**Figure 10:**
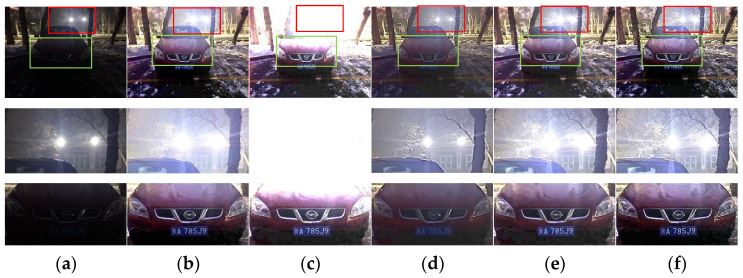
(**a**) Test image and its detail maps. The remaining images are the enhancement results and their detail maps, generated by the following methods: (**b**) ALSM, (**c**) Li et al. [26], (**d**) LR3M, (**e**) LIME, and (**f**) the proposed method.

**Figure 11:**
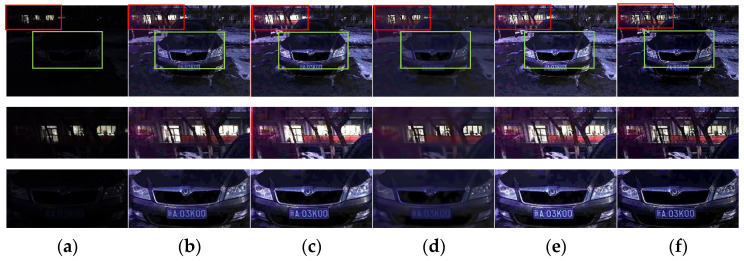
(**a**) Test image and its detail maps. The remaining images are the enhancement results and their detail maps, generated by the following methods: (**b**) ALSM, (**c**) Li et al. [26], (**d**) LR3M, (**e**) LIME, and (**f**) the proposed method.

**Figure 12:**
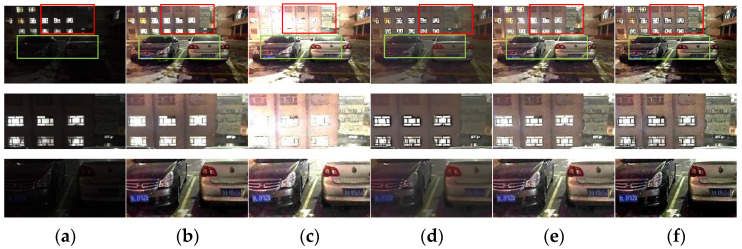
(**a**) Test image and its detail maps. The remaining images are the enhancement results and their detail maps, generated by the following methods: (**b**) ALSM, (**c**) Li et al. [26], (**d**) LR3M, (**e**) LIME, and (**f**) the proposed method.

**Figure 13:**
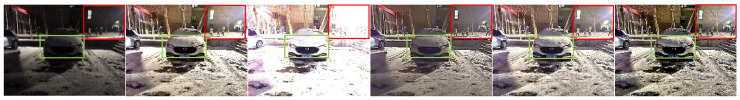

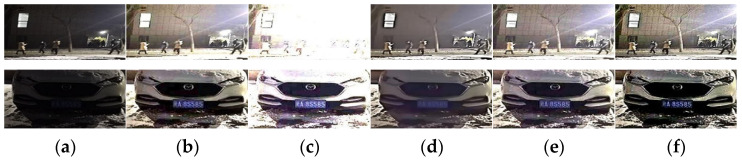
(**a**) Test image and its detail maps. The remaining images are the enhancement results and their detail maps, generated by the following methods: (**b**) ALSM, (**c**) Li et al. [26], (**d**) LR3M, (**e**) LIME, and (**f**) the proposed method.

The collected low illumination images from real scenes for experiments, as shown in Figure 10, Figure 11, Figure 12 and Figure 13, both the algorithm and the contrast algorithm can effectively improve the brightness and contrast of the test images. However, the ALSM algorithm, Li et al. [26], and the LIME algorithm are overexposed in the light source when enhancing Figure 10, making the edge of the door under the light source unclear. In images with partially illuminated areas, see Figure 11 and Figure 12. The ALSM algorithm, Li et al. [26] and the LIME algorithm do not preserve the edge details of the test images well in the lighted window area. The brightness of the enhanced images obtained by the first two algorithms in the license plate part is not as high as that obtained by the proposed algorithm. Figure 13 shows the case of bright surroundings. Li et al. [26] and the LIME algorithm can display the hidden license plate and the logo, but the background is over-enhanced. In particular, the background of the enhanced image obtained by Li et al. [26] is almost completely white. The dark area of the enhanced image obtained by the ALSM algorithm is not bright enough, and the highway edges are not clear enough due to the overexposure of the bright area. Although the LR3M algorithm does not produce the above problems in Figure 10, Figure 11, Figure 12 and Figure 13, the brightness of the enhanced image obtained by the LR3M algorithm is not bright enough, and the license plate is too blurred to see the critical information in the image. The proposed algorithm can overcome the shortcomings of the contrast algorithm and suppress overexposure to a certain extent, and the license plate is visible. The enhanced image can not only retain details and edges but also highlight the key information in the image.

#### 4.3.2. Qualitative Analysis

The different image quality evaluation indices were calculated, including average gradient (AG) [43], information entropy (IE), edge intensity (e), contrast (C), full reference image index peak signal-to-noise ratio (PSNR), patch-based contrast quality index (PCQI) [44], structural similarity (SSIM) [45]. For multi-exposure datasets, calculated the full reference and no reference image index. For images taken in the real environment, only calculated the no-reference image index. The larger the value of all the indexes, the better the enhancement effect. The time complexity of each algorithm was also recorded to better analyze the effectiveness of the algorithm. The objective evaluation of the above images is shown in Table 1, Table 2, Table 3, Table 4 and Table 5.

It can be seen from Table 1, Table 2 and Table 3 that the algorithm proposed in this paper has superior performance in 4 criteria: AG, e, C, and PCQI, and competitive performance on the other 3 criteria, i.e., for IE and SSIM it ranked third, and for PSNR it ranked fourth. Combined with subjective visual effects, it is found that the reference image is overexposed and the key information such as text is not prominent, so the PSNR and SSIM values of the proposed algorithm are slightly lower.

From Table 4 and Table 5, it can be seen that our algorithm has the highest sharpness, edge intensity, and contrast in the real image enhancement evaluation, which is higher than the second-ranked algorithm of 19.26%, 27.31%, and 14.92%, respectively. The average information entropy of this algorithm ranks second, only lower than LIME. This is because the LIME algorithm enhances the overall brightness of the image, resulting in severe over-enhancement in some areas of the image. In terms of time complexity, the proposed algorithm takes 1.68 s on average to process an image, which is relatively fast.

In summary, combining quantitative and qualitative analysis, the following conclusion can be drawn. The algorithm presented in this paper can effectively enhance the brightness and contrast of low illumination images, preserve details and edge areas, and suppress overexposure to a certain extent. Additionally, the objective indexes are better than the comparison algorithm.

## 5. Discussion

The proposed algorithm can be applied to low illumination video enhancement. For a captured video, the video is first decomposed into a single frame image sequence. Secondly, each frame image from this sequence is sequentially read to enhance this single-frame image. Considering that flickering occurs due to different average brightness of each frame image when synthesizing video, adjust the brightness of subsequent video frames based on the average brightness of the first enhanced video frame to obtain a video frame sequence with the same average brightness. Finally, the enhanced video frames are synthesized sequentially at a rate of 30 frames per second to get the final enhanced video. Through experiments on a video with a size of 1920 × 1080 and some low-illumination videos on the Internet, it is found that the proposed algorithm can be effectively used to enhance the low-illumination video.

From all experiments above, the ALSM algorithm, Li et al. [26], and LIME algorithm all overexpose an image in the bright area, resulting in loss of edge details. The LR3M algorithm produces an enhanced image with insufficient brightness and blurs key information (such as license plates, car logos, etc.) due to the addition of noise removal items. The proposed algorithm in this paper can effectively overcome the shortcomings of the above algorithms, enhancing the brightness of the image while maintaining the edges and details of the image, and suppressing overexposure to a certain extent. The reasons for the proposed algorithm being superior to the compared algorithms are as follows:

(1) A dark channel refined method is proposed to obtain a more accurate dark channel map, which can effectively enhance the brightness of the image.

(2) An anisotropic guide filter is used to refine the transmission, smooth the transmission map while preserving edges, and obtain a more detailed transmission map, which enables the enhanced image to retain the edges well.

(3) Based on the characteristics of low illumination images, an S-type function factor is defined by which the high-frequency part of the initial enhanced image is added to itself to obtain an enhanced image with more prominent details.

(4) The proposed algorithm can be effectively applied to low illumination video enhancement, controlling the average brightness of each enhanced image to reduce video flickering.

## 6. Conclusions

In this paper, detail preserving low illumination video image enhancement algorithm based on a dark channel was proposed, which uses the similarity between the inverted low illumination image and the hazy image to dehaze the inverted low illumination image. To solve the problem of insufficient brightness in the enhanced image, the initial dark channel is refined to obtain a brighter image. For missing edges and details of the image, the anisotropic guide filter is used to refine the transmission, resulting in finer transmission and less time complexity. To solve the problem of insufficient detail, the details of the initial enhanced image are optimized. Using the S-type function as the coefficient of the high frequency, the high frequency is added to the initial enhanced image to obtain the enhanced image with more details.

We described the performance of the proposed algorithm in images and videos captured in a real low illumination environment. In both quantitative and qualitative comparative analyses, the proposed algorithm is superior to state-of-the-art algorithms, which proves its effectiveness and robustness. It can be applied to different low illumination scenarios in wider computer vision applications. The limitation of the proposed algorithm is that it may amplify the noise of the image. In further, a suitable denoising algorithm can be introduced to maintain the balance between details and noise.

For video enhancement, not only can the change of pixel intensity in time be considered, but also the change of pixel intensity in space and direction can be considered to further develop low-light video enhancement, and it can be used as a preprocessing for detection and recognition to form a complete the detection and recognition system [46].

## Figures and Tables

**Table 1:** Comparison of various indexes of multi-exposure data 1 enhanced by different methods.

Method	AG	IE	e	C	PSNR	SSIM	PCQI	Times
ALSM	2.97	7.43	197.33	49.00	19.76	0.77	0.74	148.26
Li et al. [26]	2.81	5.56	245.69	46.14	12.32	0.52	0.53	1.63
LR3M	1.98	7.43	196.04	35.46	23.04	0.84	0.65	6171.03
LIME	3.15	7.58	201.14	51.09	19.96	0.78	0.73	2.37
The proposed method	4.49	7.54	386.20	69.63	20.15	0.70	0.75	6.38

**Table 2:** Comparison of various indexes of multi-exposure data 1 enhanced by different methods.

Method	AG	IE	e	C	PSNR	SSIM	PCQI	Times
ALSM	2.07	7.00	134.13	37.23	27.12	0.81	0.87	66.04
Li et al. [26]	0.91	4.65	56.64	18.13	16.85	0.50	0.59	1.60
LR3M	2.38	7.35	266.64	42.17	24.74	0.82	0.82	5017.18
LIME	2.33	7.52	148.46	41.01	21.36	0.72	0.85	2.54
The proposed method	2.94	7.39	229.70	49.21	25.61	0.77	0.95	6.55

**Table 3:** Average values of each index of 46 images in multi-exposure dataset enhanced by different methods.

Method	AG	IE	e	C	PSNR	SSIM	PCQI	Times
ALSM	2.12	7.07	118.06	36.50	22.90	0.77	0.89	121.40
Li et al. [26]	1.79	4.69	136.97	30.89	15.64	0.56	0.67	1.61
LR3M	1.54	7.27	120.64	27.58	25.53	0.81	0.81	5797.49
LIME	2.43	7.44	147.31	41.57	22.46	0.72	0.92	2.51
The proposed method	3.13	7.21	228.92	50.43	22.06	0.72	0.96	6.17

**Table 4:** Comparison of various indexes of captured data enhanced by different methods.

Data	Method	AG	IE	e	C	Times
1	ALSM	6.92	7.75	626.01	98.34	14.21
	Li et al. [26]	5.32	5.97	554.12	75.51	0.50
	LR3M	4.13	7.31	435.71	61.44	155.69
	LIME	7.80	7.73	747.25	110.03	1.04
	The proposed method	10.50	7.79	1266.27	139.26	1.77
2	ALSM	4.32	6.40	369.98	62.76	14.73
	Li et al. [26]	5.04	6.50	504.19	73.13	0.59
	LR3M	1.37	6.08	132.69	21.78	183.53
	LIME	5.78	6.98	594.95	83.49	1.05
	The proposed method	6.15	6.31	675.40	83.50	1.66
3	ALSM	5.33	7.56	500.67	81.37	15.99
	Li et al. [26]	5.57	7.40	513.40	84.73	0.62
	LR3M	3.11	6.79	544.24	50.35	171.63
	LIME	6.13	7.61	631.89	92.30	1.02
	The proposed method	7.88	7.58	930.76	114.40	1.70
4	ALSM	12.41	7.80	1635.13	158.94	15.45
	Li et al. [26]	7.51	5.01	1243.82	95.85	0.49
	LR3M	11.01	7.64	1698.36	145.12	183.92
	LIME	13.54	7.80	1791.22	171.36	1.22
	The proposed method	19.01	7.81	3101.19	226.43	1.84

**Table 5:** Comparison of objective index mean values for 100 video image enhancements by different methods.

Method	AG	IE	e	C	Times
ALSM	6.80	7.06	661.16	95.43	13.43
Li et al. [26]	7.40	6.79	921.97	104.22	0.59
LR3M	2.56	6.61	239.43	39.56	210.63
LIME	7.99	7.36	878.64	110.67	1.09
The proposed method	9.90	7.09	1268.28	130.07	1.68

## Data Availability

The algorithm performance is tested on the public data set, including https://pan.baidu.com/s/1x1Dq9xef1dBTXXHcMjPAyA (accessed on 28 July 2021). and https://ieeexplore.ieee.org/document/8237767 (accessed on 25 June 2021).
